# Intravitreal MPTP drives retinal ganglion cell loss with oral nicotinamide treatment providing robust neuroprotection

**DOI:** 10.1186/s40478-024-01782-3

**Published:** 2024-05-21

**Authors:** Anne Rombaut, Danica Jovancevic, Raymond Ching-Bong Wong, Alan Nicol, Rune Brautaset, David I. Finkelstein, Christine T. O. Nguyen, James R. Tribble, Pete A. Williams

**Affiliations:** 1 Department of Clinical Neuroscience, Division of Eye and Vision, St. Erik Eye Hospital, Karolinska Institutet, Stockholm, Sweden; 2 Centre for Eye Research Australia, Royal Victorian Eye and Ear Hospital, Melbourne, Australia; 3 Department of Surgery (Ophthalmology), The University of Melbourne, Melbourne, Australia; 4 The Florey Institute of Neuroscience and Mental Health, The University of Melbourne, Parkville, Australia; 5 Department of Optometry and Vision Sciences, The University of Melbourne, Parkville, Australia

**Keywords:** Retina, Retinal ganglion cell, Astrocyte, Microglia, Müller glia, MPTP, MPP+, Parkinson’s disease

## Abstract

Neurodegenerative diseases have common underlying pathological mechanisms including progressive neuronal dysfunction, axonal and dendritic retraction, and mitochondrial dysfunction resulting in neuronal death. The retina is often affected in common neurodegenerative diseases such as Parkinson’s and Alzheimer’s disease. Studies have demonstrated that the retina in patients with Parkinson’s disease undergoes changes that parallel the dysfunction in the brain. These changes classically include decreased levels of dopamine, accumulation of alpha-synuclein in the brain and retina, and death of dopaminergic nigral neurons and retinal amacrine cells leading to gross neuronal loss. Exploring this disease's retinal phenotype and vision-related symptoms is an important window for elucidating its pathophysiology and progression, and identifying novel ways to diagnose and treat Parkinson’s disease. 1-methyl-4-phenyl-1,2,3,6-tetrahydropyridine (MPTP) is commonly used to model Parkinson’s disease in animal models. MPTP is a neurotoxin converted to its toxic form by astrocytes, transported to neurons through the dopamine transporter, where it causes mitochondrial Complex I inhibition and neuron degeneration. Systemic administration of MPTP induces retinal changes in different animal models. In this study, we assessed the effects of MPTP on the retina directly via intravitreal injection in mice (5 mg/mL and 50 mg/mL to 7, 14 and 21 days post-injection). MPTP treatment induced the reduction of retinal ganglion cells—a sensitive neuron in the retina—at all time points investigated. This occurred without a concomitant loss of dopaminergic amacrine cells or neuroinflammation at any of the time points or concentrations tested. The observed neurodegeneration which initially affected retinal ganglion cells indicated that this method of MPTP administration could yield a fast and straightforward model of retinal ganglion cell neurodegeneration. To assess whether this model could be amenable to neuroprotection, mice were treated orally with nicotinamide (a nicotinamide adenine dinucleotide precursor) which has been demonstrated to be neuroprotective in several retinal ganglion cell injury models. Nicotinamide was strongly protective following intravitreal MPTP administration, further supporting intravitreal MPTP use as a model of retinal ganglion cell injury. As such, this model could be utilized for testing neuroprotective treatments in the context of Parkinson’s disease and retinal ganglion cell injury.

**Supplementary Information:**

The online version contains supplementary material available at 10.1186/s40478-024-01782-3.

## Introduction

Parkinson’s disease is the world’s fastest-growing neurodegenerative disease with an estimated incidence of 10 million patients worldwide in 2016, which is predicted to more than double in 2040 [9–11]. Parkinson's disease is classically characterized by a progressive decline of motor function such as bradykinesia, rest tremor and rigidity, although non-motor symptoms including vision abnormalities are increasingly well-recognized [12, 44, 47]. The motor symptoms are driven by the synaptic dysfunction and degeneration of dopaminergic neurons in the substantia nigra pars compacta leading to gross neuronal loss [16, 20, 53]. At a pathological level, Parkinson's disease is characterized by the abnormal aggregation of abnormally folded α-synuclein in neurons [24] forming Lewy bodies and Lewy neurites [24]. The disease also shares characteristics with other neurodegenerative diseases such as neuroinflammation and metabolic dysfunction [13, 23, 25, 32, 34].

Parkinson’s disease symptoms are not localized solely to the brain. Abnormalities in visual function have been demonstrated in Parkinson’s disease patients including low-contrast visual acuity, contrast sensitivity, color- and pattern-discrimination, depth and movement perception, higher-order visuospatial abilities, remodeling of the foveal pit, retinal thinning on optical coherence tomography scans and an abnormal electroretinogram [4, 37]. Supporting this, intraretinal α-synuclein aggregates have been identified in inner retinal neurons in post-mortem Parkinson's disease retinas [4]. Similar to the brain, a loss of retinal dopaminergic neurons has been identified (dopaminergic amacrine cells) [40]. Optical coherence tomography (OCT) imaging has demonstrated that Parkinson's disease patients have a significantly thinner retinal nerve fiber layer, ganglion cell layer and inner plexiform layer compared to healthy controls [8, 37, 21, 63]. This is further supported by studies demonstrating that patients with a lower ganglion cell-inner plexiform layer thickness and peripapillary retinal nerve fiber layer thickness have significantly increased risk of cognitive decline at 3 years after experiment enrollment with significant associations between retinal thickness and motor dysfunction [37]. This highlights the potential benefits of early detection and understanding of Parkinson’s disease by studying the retina.

A commonly used inducible model of Parkinson's disease is the systemic delivery of the neurotoxin 1-methyl-4-phenyl-1, 2, 3, 6-tetrahydropyridine (MPTP) which results in the acute degeneration of dopaminergic neurons and a recapitulation of the severe physical symptoms seen in Parkinson's disease [52]. It is important to remember that this toxin was discovered to produce a high-fidelity phenocopy of idiopathic Parkinson’s disease by young drug addicts who unknowingly injected themselves with MPTP (Langston, 2017). MPTP is a lipophilic molecule that crosses the blood–brain barrier, is readily taken up by glial cells (predominantly astrocytes) and metabolized by the enzyme monoamine oxidase B (MAO-B) to produce 1-methyl-4-phenylpyridinium (MPP +) [52]. MPP+ is released by glia into the extracellular space and taken up by dopaminergic neurons through dopamine transporters (*Slc6a3*), which are expressed on and near the dopaminergic synapses [5]. Once inside the dopaminergic neurons, MPP+ enters the mitochondria where it inhibits predominantly Complex I [29, 39], and to a lower degree Complexes III and IV of the electron transport chain [35]. This inhibition results in a decrease in adenosine triphosphate (ATP) production, paired with an increase in the production of reactive oxygen species resulting in the death of the metabolically vulnerable neurons.

The systemic administration of MPTP results in a retinal phenotype in non-human primates [15, 18, 19], rabbits [26, 69], and rodents [17, 54]. Retinal dopamine levels decrease as early as 7 days after systemic MPTP administration [54, 69] and pending the animal model and dose employed, alterations in the electroretinogram corresponding to amacrine cells (oscillatory potentials), photoreceptors (a-wave) and bipolar cells (b-wave) have been reported. Forty-five days after systemic MPTP injection in mice, dopaminergic amacrine cell numbers were significantly reduced by 9%, oscillatory potential peak amplitude time was significantly delayed by 7–13% and the outer plexiform layer was significantly thinned. The treatment of these mice with L-DOPA ameliorated the delay in oscillatory potential but did not modify the survival of dopaminergic amacrine cells or the thickness of the outer plexiform layer [55].

The mechanisms of altered vision in Parkinson's disease are currently being hotly debated. A model that generates a retinal phenotype in the absence of motor symptoms would be an important tool to decipher retinal changes in Parkinson’s disease.

## Materials and methods

### Animal strain and husbandry

All breeding and experimental procedures were managed following the Association for Research for Vision and Ophthalmology Statement for the Use of Animals in Ophthalmic and Research. Individual study protocols were accepted by Stockholm’s Committee for Ethical Animal Research (3909–2023). Animals were housed in a regulated environment, (12 h light/12 h dark cycle) and fed with food and water ad libitum. C57BL/6 J and B6.Cg-Tg(Thy1-cyan fluorescent protein (CFP))23Jrs/J (JAX stock number #003710; ~ 80% of retinal ganglion cells are CFP+ [66], see Supplementary material) mouse strains were purchased from The Jackson Laboratory (Bar Harbor, ME, USA) and bred and maintained in-house. Both male and female mice in equal numbers were used at 12–20 weeks of age. For animal groups that were treated with nicotinamide (NAM), NAM was dissolved in drinking water to achieve a dose of ~ 500 mg/kg/d (based on average water consumption) starting 7 days before MPTP injection and ending at day 21 post-injection. Water was protected from light and changed every 3–5 days. A complete list of *n* of all mice/samples is shown in Table 1.

**Table 1:** Group size per condition and experiment

Experiment	*n* Control		*n* MPTP 5 mg/mL				*n* MPTP 50 mg/mL			
	naive	d21 (vehicle)	d7	d14	d21	d21(NAM)	d7	d14	d21	d21 (NAM)
TH+ cell										
Counts	8	11	7	6	7	–	7	5	5	–
Dendritic reconstruction	5	11	7	6	7	–	7	5	5	–
RBPMS+ retinal ganglion cell counts	7	9	9	6	6	5	7	5	9	7
CFP+ retinal ganglion cell counts (see supplementary material)	7	6	9	6	6	5	6	5	7	7
Layer thickness	–	8	12	–	–	–	6	–	–	–
Other immuno-fluorescent labelling										
Prox1	–	8	9	–	–	–	5	–	–	–
IBA1	–	8	10	–	–	–	6	–	–	–
GFAP	–	8	10	–	–	–	6	–	–	–
GS	–	8	10	–	–	–	6	–	–	–

### Gene expression analysis

To identify the expression of *Slc6a3* and *Maob* in mouse retina, single-cell RNA-sequencing data from Macosko et al. [33] was analyzed using Spectacle [61]. Heatmaps and violin plots were generated from the original cell clusters identified in Macosko et al. [33]. To confirm whether MAOB expression also occurred in human retinal ganglion cells, MAOB was analyzed in single-cell (sc) RNAseq (Yan et al. [70]) and in single-nucleus (sn) RNAseq (Liang et al. [31]). The datasets were processed using Seurat V3.2. retinal ganglion cell types were grouped according to the clustering and cell annotations provided by the authors. To compare MAOB expression across individual retinal ganglion cells, the NormalizeData() function was used to generate normalized and log-transformed single cell expression.

### Intravitreal MPTP model

MPTP (MPTP hydrochloride, SelleckChem) was dissolved in Hank’s Balanced Salt Solution (1 × HBSS, Gibco) to 5 mg/mL and 50 mg/mL guided by previous studies using intravitreal delivery in the goldfish [43, 59, 60]. Mice were anaesthetized with an intraperitoneal injection of ketamine (37.5 mg/kg) and medetomidine hydrochloride (1.25 mg/kg). A volume of 2 µL of MPTP or HBSS (vehicle only control) was injected intravitreally using a 35G tri-beveled needle (NanoFil, WPI) attached to a Hamilton syringe. Mice received either bilateral MPTP (5 or 50 mg/mL), bilateral HBSS or remained as bilateral naïve controls. At 7, 14 or 21 days following injection, mice were euthanized by cervical dislocation, their eyes were enucleated and fixed for 2 h by submersion in 3.7% paraformaldehyde (PFA 37%, Fisher BioReagents) in HBSS.

### Immunofluorescence antibody labeling of dopaminergic amacrine cells and retinal ganglion cells

Retinas were dissected free from globes and transferred to slides as flat-mounts. Retinas were permeabilized with 0.5% Triton X (VWR Chemicals) in 1 × Phosphate-Buffered Saline (PBS) for 30 min at room temperature, incubated with 1% Bovine Serum Albumin (BSA, Fischer Scientific) in HBSS for 30 min at room temperature and incubated with either anti-tyrosine hydroxylase (TH) primary antibody (1:500, AB152, EMD Millipore) or anti- RNA-binding protein with multiple splicing (RBPMS, a selective marker of retinal ganglion cells [49])- antibody (0.002 mg/mL NBP2-20112, Novus Biologicals) and polyclonal chicken anti-green fluorescent protein (GFP) antibody (0.02 mg/mL, AB13970, Abcam) overnight at 4 °C. Retinas were washed 5 times in PBS for 5 min and a secondary antibody was applied for 4 h at room temperature. Secondary antibodies were either Alexa Fluor 568 goat anti-rabbit (0.004 mg/mL, A11011, Invitrogen), or Alexa Fluor 568 goat anti-rabbit and Alexa Fluor 488 fluorophore goat anti-chicken (0.004 mg/mL, A11039, Invitrogen) in combination. After the incubation, retinas were washed again five times in PBS and ToPro3 iodide nuclear stain (0.671 mg/mL, T3605, Invitrogen) was applied for 10 min. Retinas were then mounted using Fluoromount-G mounting media (Invitrogen) and coverslips. Slides were sealed with nail varnish and were stored at 4 °C before imaging.

### Microscopy and image analysis of dopaminergic amacrine cells and retinal ganglion cells

High-resolution images of anti-TH labelled dopaminergic amacrine cell dendritic fields were acquired using a Zeiss LSM800-Airy CLSM confocal microscope. Two images were acquired per retina at ± 1000 μm centered around the optic nerve head*.* The images were acquired at 20 × magnification (numerical aperture 0.8, imaging area: 319.45(*x*) × 319.45(*y*) × ~ 14(*z*) μm; pixel resolution: 0.17(*x*) × 0.17(*y*) × 0.62(*z*) μm, dry objective). All imaging parameters were kept constant. Imaris Software (v. 9.2.1, Bitplane) was used to reconstruct the dendritic fields of the TH+ cells. 3D reconstructions were built with the Filament tracer option for the whole channel, with a constant cell soma diameter, thinnest dendrite diameter, and seed point threshold set. The dendrite varicosities were built using the Spots option for the whole channel, allowing for spots with different sizes. To specifically select only spots associated with the reconstructed dendrites (representing varicosities), the Find spots close to filaments tool was used with a fixed distance between the spot and the filaments to select only spots within the filament volume. The dendrites and varicosities were reconstructed for the whole image area (319.45(*x*) × 319.45(*y*) × ~ 14(*z*) μm). From these reconstructions, the dendrite length and volume, and varicosities count and volume were extracted. To quantify the density of TH+ amacrine cell somas, images were acquired on a Leica DMi8 microscope with a CoolLED pE-300 white LED-based light source and a Leica DFC7000 T fluorescence color camera (all Leica). Six images per retina at 20 × magnification (numerical aperture 0.4, imaging area: 665.28 × 665.28 μm, dry objective) were acquired equidistantly at 0, 2, 4, 6, 8 and 10 o’clock from a superior to inferior line through the optic nerve head at an eccentricity of roughly 1000 μm. Cell counting was performed in Fiji (v. 2.3.0) using the Cell counter plugin from the whole image area (665.28 × 665.28 μm) and subsequently normalized to 0.1 mm^2^ area. Retinal ganglion cell density was analyzed similarly using the Leica DMi8, with 6 images per retina acquired as above but at 40 × resolution (numerical aperture 0.55, imaging area: 332.8 × 332.8 µm, dry objective). Images were cropped to 100 × 100 μm before counting as above. RBPMS+ cells and 4′,6-diamidino-2-phenylindole (DAPI) nuclei (only round nuclei were considered, thus discarding vascular endothelium) were counted. The mean of cell counts per retina was measured across the 6 images and expressed as a density per 0.01 mm^2^.

### Cryo-sectioning and analysis of neurodegeneration and neuroinflammation

Following fixation in PFA, eyes were cryopreserved through a sucrose gradient of 10%, 20% and 30% over two days. Eyes were then frozen in optimal cutting temperature medium (Sakura) on dry ice and stored at − 80 °C. Cryo-sections were cut on a cryostat (Cryostar NX70, Thermo Scientific) at 20 μm thickness (anterior to dorsal plane) and collected all in the same orientation on Superfrost slides before storage at − 20 °C. Slides were warmed to room temperature and post-fixed with 3.7% PFA for 10 min, before following the same immunolabelling protocol as above with the following exceptions. Primary antibodies used were anti-prospero-related homeobox 1 (Prox1) antibody (1:1000, 925202, Biolegend), anti-ionized calcium-binding adaptor molecule 1 (IBA1) primary antibody (0.002 mg/mL, AB178846, Abcam), anti- glial fibrillary acidic protein (GFAP) primary antibody (1:500, NBP1-05198, Novus Biologicals) or anti-glutamine synthetase (GS) primary antibody (1:1000, NBP110-41404; Novus Biologicals). Secondary antibodies used were Alexa Fluor 568 goat anti-chicken (0.004 mg/mL, A11041, Invitrogen) or Alexa Fluor 568 goat anti-rabbit (0.004 mg/mL, A11011, Invitrogen).

For the pan-amacrine cell marker Prox1, images were acquired on a Zeiss LSM 800 confocal microscope with a 5 × and 20 × objective. For 5 × images, one image was acquired per section, centered around the optic nerve head, and two sections were imaged per eye. Images were Z-stacks (2 µm slices over a total thickness of 14 µm to collect all signal over the whole INL). The size of the image captured was 1277.8 µm × 1277.8 µm (1024 × 1024 px), covering on average 60% of the average mouse retina. Imaging parameters were kept constant. For the sample size, see Table 1. For 20 × images, two images were taken per section, at ± 1000 µm centered around the optic nerve head, and one section was imaged per eye. Images were Z-stacks (2 µm slices over a total thickness of 14 µm to collect all signal over the whole INL). The size of the images captured was 319.45 × 319.45 µm (1024 × 1024 px), covering on average 13% of the average mouse retina. Imaging parameters were kept constant within antibody conditions. For the sample size, see Table 1. Images for Prox1 were analyzed in Fiji. The Z-stacks were compressed retaining maximum intensity, and thresholded to exclude background signal (from secondary antibody only control sections), keeping the threshold constant for all the images. 5 × images were used to assess gross Prox1 labelling. After selecting the inner retina, guided by the nuclear labelling channel, the average pixel intensity (mean gray value) was measured. In 20 × images, all INL nuclei and Prox1+ cells were counted using the Cell counter tool. Both the mean gray values and counts were averaged per eye.

For the analysis of the neuroinflammatory markers IBA1, GFAP and GS, images were acquired as Z-stacks on a Zeiss LSM 800 confocal microscope with a 5 × objective as described for the Prox1 intensity analysis above (total depth of Z-stacks: IBA1 = 34 µm, GFAP = 34 µm, GS = 26 µm to capture all signal adequately), and the images were z-compressed and thresholded as described above for Prox1 analysis. The percentage of inner retina (inner limiting membrane to INL lower border) covered by the glial markers (percentage (of selected) area) was calculated using the Analyze particles tool. The percentage area values were averaged per eye and plotted.

For the analysis of retinal layer thickness, images were acquired on a Leica DMi8 (as above) with a 20 × objective. Two images were taken per section, at ± 500 µm centered around the optic nerve head, and one section was imaged per eye. Images were Z-stacks (1.214 µm slices over a total thickness of 23 µm to collect the whole INL). The size of the images captured was 332.8 × 332.8 µm (2048 × 2048 px), covering on average 13% of the average mouse retina. Imaging parameters were kept constant within antibody conditions. For the sample size, see Table 1. The thickness of the retinal layers at ± 500 μm centered around the optic nerve head was measured using the Line tool in Fiji. For each image, the thickness of each layer (nerve fiber layer = NFL, ganglion cell layer = GCL, inner plexiform layer = IPL, inner nuclear layer = INL, outer plexiform layer = OPL, outer nuclear layer = ONL) was measured at the image edges and center and the average of these 3 measurements was calculated. These values were then averaged per eye and plotted.

### Statistical analysis

Statistical analysis was performed in R using R Studio. A *Shapiro Wilk* test was used to test the normality of the data. One-way *ANOVA* followed by *Dunnett’s multiple comparison *post hoc test was applied appropriately to analyze normally distributed data. *Kruskal–Wallis one way analysis of variance* followed by *pairwise Wilcoxon rank sum* test was used to analyze non-normally distributed data. For these non-parametric tests no 95% confidence intervals (CI) were reported. Differences with *P* < 0.05 were considered significant. * = *P* < 0.05, ** = *P* < 0.01, *** = *P* < 0.001. For the box plots, the median is represented by the center hinge with lower and upper hinges indicating the first and third quartiles, whereas whiskers denote 1.5 times the interquartile range.

## Results

### Intravitreal MPTP administration does not result in TH+ cell loss

**Fig. 1:**
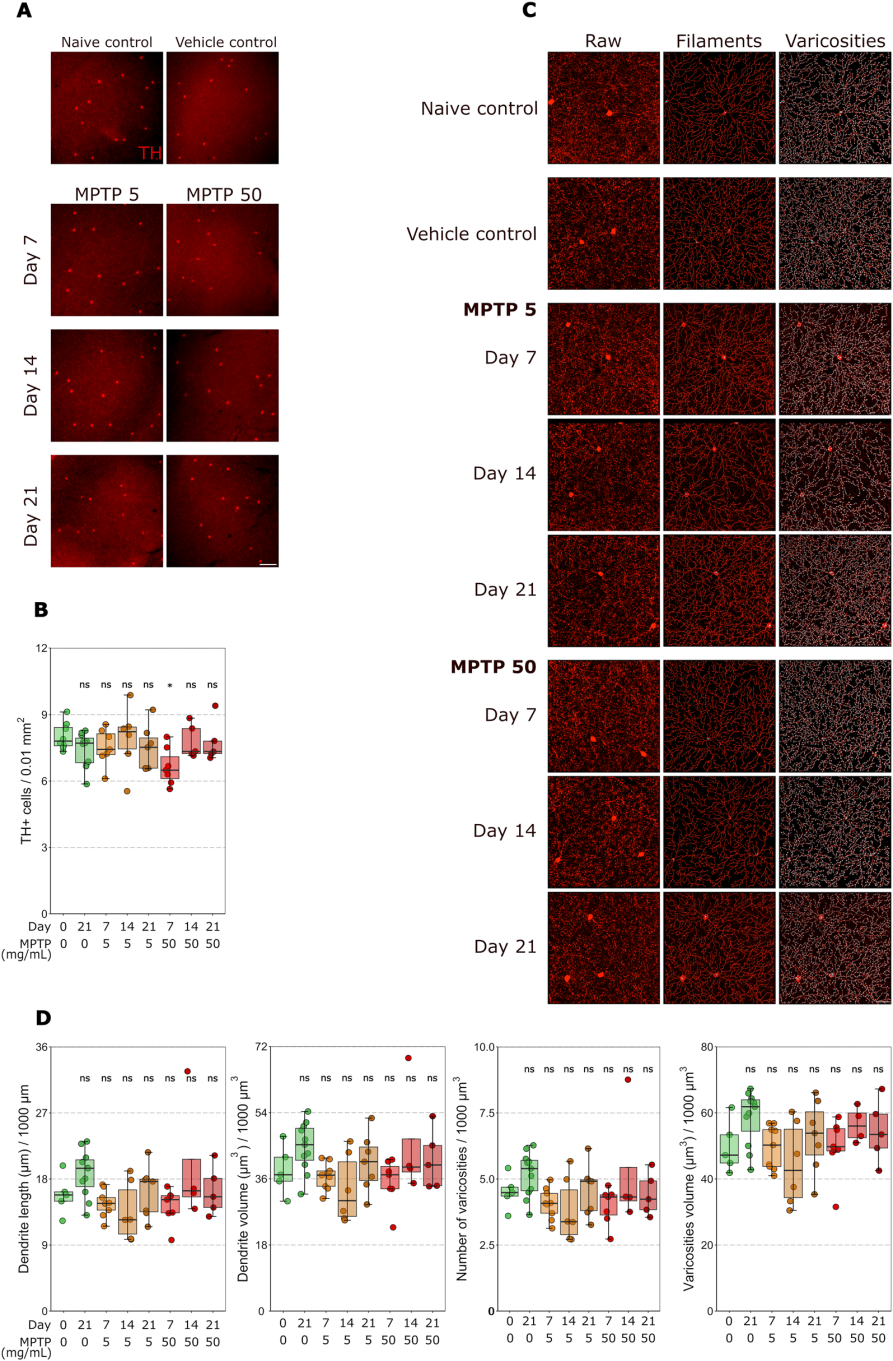
Intravitreal MPTP administration does not result in TH^+^ cell loss. **A** Dopaminergic amacrine cells were labelled with anti-TH antibodies in whole-mounted retinas. **B** The density of TH+ cells was not significantly different between the naïve and vehicle control (green) groups and between the vehicle control and the groups injected with 5 mg/mL MPTP (orange) or 50 mg/mL MPTP (red) at 7, 14, or 21 days post-injection. **C** The dendritic fields of the TH+ amacrine cells were reconstructed using Imaris. **D** From the reconstructions, the total dendrite length, total dendrite volume, number of varicosities, and total varicosity volume were quantified. There are no significant differences between the naïve and vehicle control (green) groups, the groups injected with 5 mg/mL MPTP (orange), or 50 mg/mL MPTP (red) at 7, 14 and 21 days post-injection for all three of these parameters supporting a lack of dendritic changes. Scale bar = 100 µm in A and C

Dopaminergic amacrine cell loss is a key retinal phenotype in Parkinson’s disease. To investigate whether the intravitreal administration of MPTP results in loss of dopaminergic neurons, we quantified the density of TH-positive dopaminergic amacrine cells at 7, 14 and 21 days post MPTP injection (5 or 50 mg/mL) (Fig. 1A).

There was no significant difference in TH+ neuronal soma counts between naïve controls and vehicle (HBSS) injected controls at 21 days post-injection (Table 2; Fig. 1B). There was no significant difference in the density of TH+ cells at any time point for either dose of MPTP compared to naïve controls, except for the 50 mg/ml dose 7 days after injection (Table 2; Fig. 1B). Since dendritic integrity offers a more nuanced reflection of cell health than absolute cell soma loss, we reconstructed and analyzed the morphology of TH+ dendrites (Fig. 1C). Given the overlapping tiling of the TH+ cell dendritic fields, we reconstructed and analyzed TH+ dendrites and dendrite varicosities within a field of view and assessed variables that reflect the integrity of that dendrite network. We measured the length and volume of connected dendrites and from these calculated a mean length and volume per retina per condition. There were no significant differences between vehicle and naïve controls for dendrite length or volume (Table 2; Fig. 1D). There were no significant differences for dendrite length or volume at any time point for either dose of MPTP compared to naïve controls (Table 2; Fig. 1D). There were no significant differences between vehicle and naïve controls for varicosities count or volume (Table 2; Fig. 1D). Additionally, there were no significant differences for varicosities count or volume at any time point for either dose of MPTP compared to naive controls (Table 2; Fig. 1D). Together, these data indicate that intravitreal injection of MPTP up to 50 mg/mL does not result in TH+ cell soma or integrity loss up to 21 days post-injection.

**Table 2:** *P* value and 95% CI per comparison of conditions in the TH+ cell analysis

TH+ cell analysis	*P* value	95% CI
*Cell soma count*		
Naïve control—vehicle control	0.600	[− 1.738, 0.555]
Naïve control—MPTP 5 mg/ml 7 days after injection	0.853	[− 1.760, 0.794]
Naïve control—MPTP 5 mg/ml 14 days after injection	1.000	[− 1.421, 1.244]
Naïve control—MPTP 5 mg/ml 21 days after injection	0.777	[− 1.819, 0.735]
Naïve control—MPTP 50 mg/ml 7 days after injection	0.031 *	[− 2.647, − 0.092]
Naïve control—MPTP 50 mg/ml 14 days after injection	0.998	[− 1.639, 1.174]
Naïve control—MPTP 50 mg/ml 21 days after injection	0.997	[− 1.658, 1.155]
*Dendrite length*		
Naïve control—vehicle control	0.680	–
Naïve control—MPTP 5 mg/ml 7 days after injection	0.710	–
Naïve control—MPTP 5 mg/ml 14 days after injection	0.710	–
Naïve control—MPTP 5 mg/ml 21 days after injection	0.980	–
Naïve control—MPTP 50 mg/ml 7 days after injection	0.710	–
Naïve control—MPTP 50 mg/ml 14 days after injection	0.760	–
Naïve control—MPTP 50 mg/ml 21 days after injection	1.000	–
*Dendrite volume*		
Naïve control—vehicle control	0.640	–
Naïve control—MPTP 5 mg/ml 7 days after injection	0.890	–
Naïve control—MPTP 5 mg/ml 14 days after injection	0.640	–
Naïve control—MPTP 5 mg/ml 21 days after injection	0.960	–
Naïve control—MPTP 50 mg/ml 7 days after injection	0.780	–
Naïve control—MPTP 50 mg/ml 14 days after injection	0.890	–
Naïve control—MPTP 50 mg/ml 21 days after injection	0.960	–
*Varicosities count*		
Naïve control—vehicle control	0.700	–
Naïve control—MPTP 5 mg/ml 7 days after injection	0.700	–
Naïve control—MPTP 5 mg/ml 14 days after injection	0.700	–
Naïve control—MPTP 5 mg/ml 21 days after injection	0.970	–
Naïve control—MPTP 50 mg/ml 7 days after injection	0.700	–
Naïve control—MPTP 50 mg/ml 14 days after injection	0.930	–
Naïve control—MPTP 50 mg/ml 21 days after injection	0.970	–
*Varicosities volume*		
Naïve control—vehicle control	0.289	[− 4131.158, 21,778.773]
Naïve control—MPTP 5 mg/ml 7 days after injection	1.000	[− 13670.351, 13,124.131]
Naïve control—MPTP 5 mg/ml 14 days after injection	0.839	[− 19863.108, 9225.526]
Naïve control—MPTP 5 mg/ml 21 days after injection	0.983	[− 10919.090, 17,209.257]
Naïve control—MPTP 50 mg/ml 7 days after injection	1.000	[− 14200.632, 13,927.715]
Naïve control—MPTP 50 mg/ml 14 days after injection	0.759	[− 9469.938, 22,755.132]
Naïve control—MPTP 50 mg/ml 21 days after injection	0.920	[− 10529.049, 19,853.038]

Significance: *P < 0.05, **P < 0.01, ***P < 0.001

### Intravitreal MPTP administration drives retinal ganglion cell loss

**Fig. 2:**
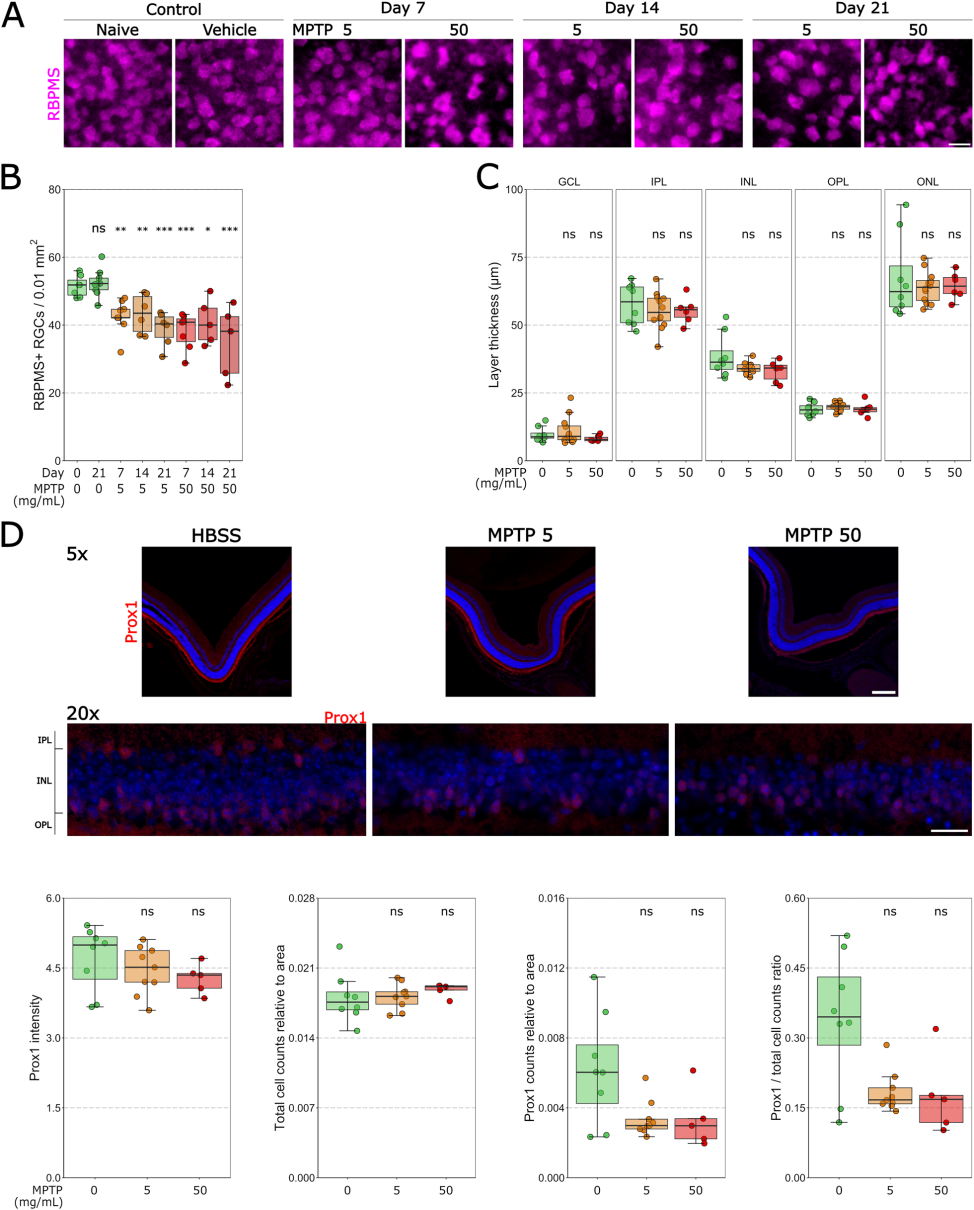
Intravitreal MPTP administration drives retinal ganglion cell loss. **A** Retinal ganglion cells were fluorescently labelled in whole-mounted retina with anti-RBPMS (magenta). **B** There were no significant differences between the naïve and vehicle control (green) groups. RBPMS density significantly decreased relative to the vehicle control at 7, 14 and 21 days post-injection for 5 mg/mL (orange) and 50 mg/mL (red) MPTP. **C** The thickness of all retinal layers (GCL, IPL, INL, OPL, and ONL) was measured from cross-sections. For all retinal layers, there were no significant differences between the vehicle control (green) and 5 mg/mL MPTP (orange) or 5 mg/mL MPTP (red) at 7 days post MPTP injection. **D** All amacrine cells were fluorescently labelled in retinal cross-sections with anti-Prox1 antibodies. Images captured with a 5 × objective were analyzed for Prox1 signal intensity, images captured with a 20 × objective were analyzed for Prox1+ cell soma counts. This data was plotted. There were no significant differences in signal intensity between vehicle control (green) and 5 mg/mL MPTP (orange) or 50 mg/mL MPTP (red). The number of Prox1+ cells, the total number of INL cells (relative to the measured INL area), and the relative density of Prox1+ cells (Prox1+ cells/total cell number) did not significantly change over the different conditions. There were no significant differences in between vehicle control (green) and 5 mg/mL MPTP (orange) or 50 mg/mL MPTP (red). Scale bar = 20 µm in A and D (20 ×), 200 µm in D (5 ×)

MPTP is a neurotoxin affecting Complex I. Since retinal ganglion cells are particularly vulnerable to metabolic stress [36, 68], we next investigated retinal ganglion cell viability. To empirically test this, Thy1-CFP mice (~ 80% of retinal ganglion cells are CFP+ [66], see supplementary material), underwent the MPTP injection paradigm as above. The density of RBPMS+ and CFP+ retinal ganglion cells was quantified (Fig. 2A, Additional file 1: Fig. S1A). There was no change in RBPMS+ or CFP+ retinal ganglion cell density between naïve controls and vehicle controls at 21 days post-injection, supporting the absence of neurodegeneration and loss of retinal ganglion cells from the injection procedure (Table 3, Fig. 2B; Additional file 1: Table S1, Fig. S1B). RBPMS+ retinal ganglion cell density was significantly reduced following the injection of both doses of MPTP on 7, 14 and 21 days after injection. At a dose of 5 mg/mL of MPTP, significant loss of RBPMS+ and CFP+ density occurred as early as 7 days post-injection (Table 3, Fig. 2B; Additional file 1: Table S1, Fig. S1B) and remained consistent to 14 days (Table 3, Fig. 2B; Additional file 1: Table S1, Fig. S1B) and 21 days post-injection (Table 3, Fig. 2B; Additional file 1: Table S1, Fig. S1B) compared to the naïve control samples. This trend was repeated for 50 mg/mL of MPTP at 7 (Table 3, Fig. 2B; Additional file 1: Table S1, Fig. S1B), 14 (Table 3, Fig. 2B; Additional file 1: Table S1, Fig. S1B) and 21 days (Table 3, Fig. 2B; Additional file 1: Additional file 1: Table S1, Fig. S1B) post-injection. Intravitreal injection of MPTP therefore drives a robust loss of retinal ganglion cells.

**Table 3:** *P* value and 95% CI per comparison of conditions in the retinal ganglion cell analysis

Retinal ganglion cells	*P* value	95% CI
RBMPS+ cell soma count		
Naïve control—vehicle control	1.000	[− 6.708, 8.691]
Naïve control—MPTP 5 mg/ml 7 days after injection	0.002**	[− 14.718, − 3.028]
Naïve control—MPTP 5 mg/ml 14 days after injection	0.010**	[− 14.603, − 1.699]
Naïve control—MPTP 5 mg/ml 21 days after injection	< 0.001***	[− 19.019, − 6.116]
Naïve control—MPTP 50 mg/ml 7 days after injection	< 0.001***	[− 21.536, − 5.116]
Naïve control—MPTP 50 mg/ml 14 days after injection	0.018*	[− 19.525, − 1.537]
Naïve control—MPTP 50 mg/ml 21 days after injection	< 0.001***	[− 25.325, 7.337]

Significance: *P < 0.05, **P < 0.01, ***P < 0.001

**Fig. 3:**
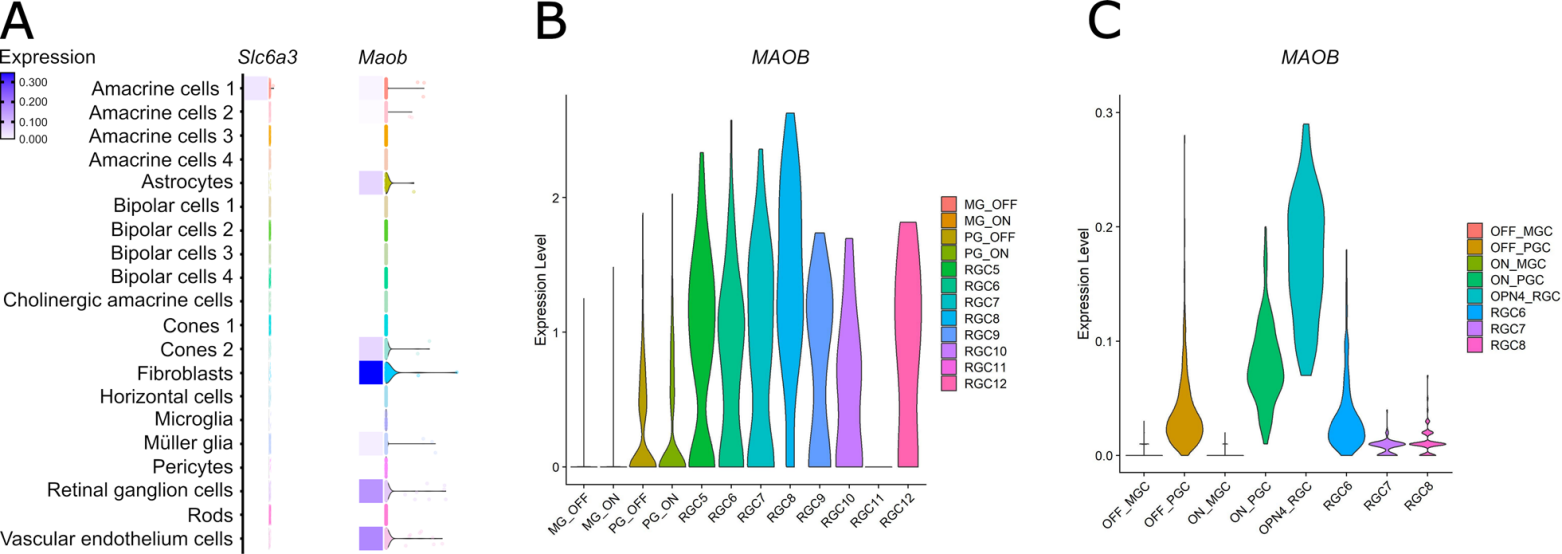
Retinal ganglion cells express *MAOB*. **A** The expression levels of the *Slc6a3* (encodes the dopamine transporter protein) and *Maob* (encodes the monoamine oxidase B protein) genes were explored in single-cell RNA-sequencing data from mouse retina. *Slc6a3* is only expressed in amacrine cell cluster 1 (likely containing dopaminergic amacrine cells) suggesting that other retinal cells do not uptake MPP+ . *Maob* is most abundantly expressed in fibroblasts, retinal ganglion cells and vascular endothelium cells, suggesting a direct route for retinal ganglion cells to internally process MPTP to MPP+. This is supported by expression levels of MAOB in **B** single-cell RNA-sequencing and **C** single-nucleus RNA sequencing data from human retina, showing that MAOB is expressed in multiple retinal ganglion cell clusters

As we identified an unexpectedly large loss of retinal ganglion cells, we next assessed gross retinal structure by measuring the thickness of individual retinal layers (Fig. 2C). At 7 days post-injection, there were no significant changes in retinal thickness between vehicle and 5 or 50 mg/mL MPTP in the GCL (Table 4; Fig. 2C), IPL (Table 4; Fig. 2C), INL (Table 4; Fig. 2C), OPL (Table 4; Fig. 2C), or ONL (Table 4; Fig. 2C) supporting a lack of a gross retinal degenerative phenotype and rather a specific loss of retinal ganglion cells. Supporting this, there was no significant loss of Prox1 signal intensity (pan amacrine cell marker; Fig. 2D) or Prox1+ cell count relative to the total cell number (Table 4; Fig. 2D) labelling in the retina at 7 days post-injection relative to vehicle controls. Both the number of Prox1+ cells and total number of cells did not significantly change over the different conditions, relative to the measured INL area (statistics not shown, Fig. 2D). Collectively, these data demonstrate that MPTP drives the selective loss of retinal ganglion cells in the absence of degeneration of amacrine cells or gross-retinal neurodegeneration. To understand why retinal ganglion cells, which are not dopaminergic, degenerate in response to MPTP we queried gene expression of the enzymes/transporters involved in the processing and uptake of MPTP from publicly available RNA-sequencing of mouse and human retina. As expected, expression of *Slc6a3* (encoding DAT, the dopamine transporter) in the mouse retina is negligible in all retinal cell types except for a cluster representing a subset of amacrine cells (Fig. 3) which likely contains dopaminergic amacrine cells. Retinal ganglion cells are therefore unlikely to be taking up MPP+ from the extracellular space. However, *Maob* (encoding the enzyme MAO-B) is predominantly expressed by retinal astrocytes, fibroblast, vascular endothelium and retinal ganglion cells in mouse retina (Fig. 3A). In the human retina, single-cell and single nucleus RNA sequencing identified multiple clusters of retinal ganglion cells expressing *MAOB* (Fig. 3C and D). These results both in mouse and human retina support the potential for retinal ganglion cells to directly uptake the membrane-permeable MPTP and process it internally to MPP+ where it could initiate degeneration.

**Table 4:** *P* value and 95% CI per comparison of conditions in the retinal layer thickness analysis

Retinal layer thickness	*P* value	95% CI
GCL		
Vehicle control—MPTP 5 mg/ml	0.970	–
Vehicle control—MPTP 50 mg/ml	0.420	–
IPL		
Vehicle control—MPTP 5 mg/ml	0.610	[− 9.757, 4.591]
Vehicle control—MPTP 50 mg/ml	0.729	[− 10.905, 6.072]
INL		
Vehicle control—MPTP 5 mg/ml	0.118	[− 9.734, 0.984]
Vehicle control—MPTP 50 mg/ml	0.090	[− 11.896, 0.785]
OPL		
Vehicle control—MPTP 5 mg/ml	0.609	[− 1.425, 3.036]
Vehicle control—MPTP 50 mg/ml	0.989	[− 2.201, 2.778]
ONL		
Vehicle control—MPTP 5 mg/ml	0.850	–
Vehicle control—MPTP 50 mg/ml	0.850	–
Prox1 intensity		
Vehicle control—MPTP 5 mg/ml	0.350	–
Vehicle control—MPTP 50 mg/ml	0.350	–
Prox1+ cell soma count		
Vehicle control—MPTP 5 mg/ml	0.110	–
Vehicle control—MPTP 50 mg/ml	0.110	–

### No detectible early retinal neuroinflammation following intravitreal MPTP administration

**Fig. 4:**
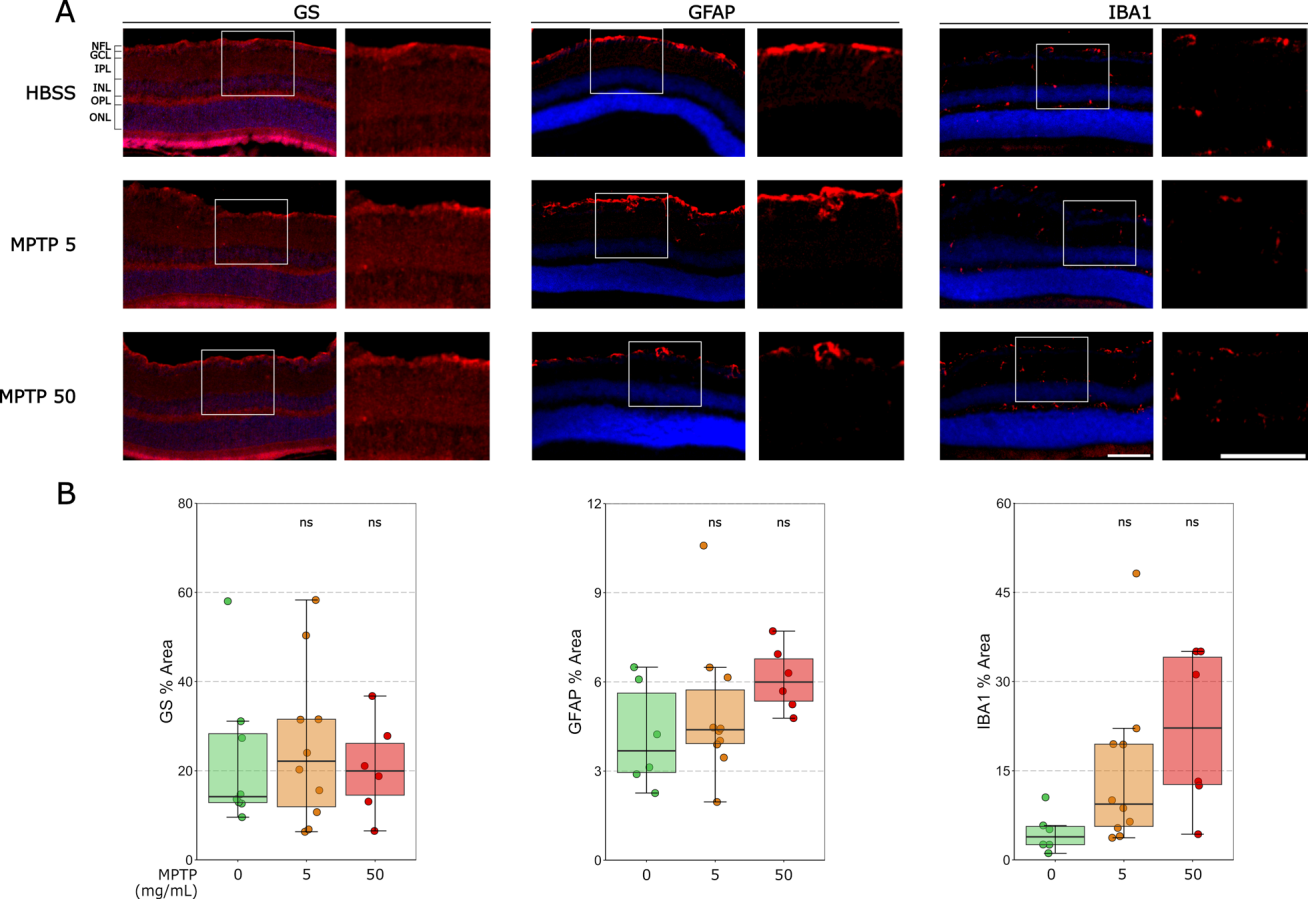
Intravitreal MPTP administration does not drive an early retinal neuroinflammatory phenotype. **A** Microglia were labelled with anti-IBA1, astrocytes and Müller cells with anti-GFAP and Müller cells with anti-GS in retina cross-sections. **B** For IBA1, GFAP and GS, there were no significant differences in percentage coverage of the inner retina between the vehicle control (green) and 5 mg/mL MPTP (orange) or 50 mg/mL MPTP (red). Scale bar = 100 µm

Neuroinflammation is a key component of Parkinson’s disease [3]. To investigate whether intravitreal MPTP injection results in retinal neuroinflammation, we labeled IBA1 (microglia/infiltrating macrophage marker), GFAP (astrocyte/ Müller cell marker) and GS (Müller cell marker) 7 days after MPTP injection (Fig. 4A). In comparison to vehicle-injected controls, there was no significant change in the percentage of the inner retina occupied by IBA1 (Table 5,Fig. 4B), GFAP (Table 5; Fig. 4B) and GS (Table 5; Fig. 4B) labelling. This supports the absence of a gross neuroinflammatory phenotype in the inner retina following MPTP intravitreal injection.

**Table 5:** *P* value and 95% CI per comparison of conditions in the inflammatory marker analysis

Neuroinflammatory markers	*P* value	95% CI
IBA1 intensity		
Vehicle control—MPTP 5 mg/ml	0.41	–
Vehicle control—MPTP 50 mg/ml	0.12	–
GFAP intensity		
Vehicle control—MPTP 5 mg/ml	0.104	[− 0.377, 4,444]
Vehicle control—MPTP 50 mg/ml	0.095	[− 0.374, 5,115]
GS intensity		
Vehicle control—MPTP 5 mg/ml	0.113	[− 2.421, 25.370]
Vehicle control—MPTP 50 mg/ml	0.110	[− 2.673, 28.969]

### Nicotinamide provides robust, long-term retinal ganglion cell neuroprotection following intravitreal MPTP administration

**Fig. 5:**
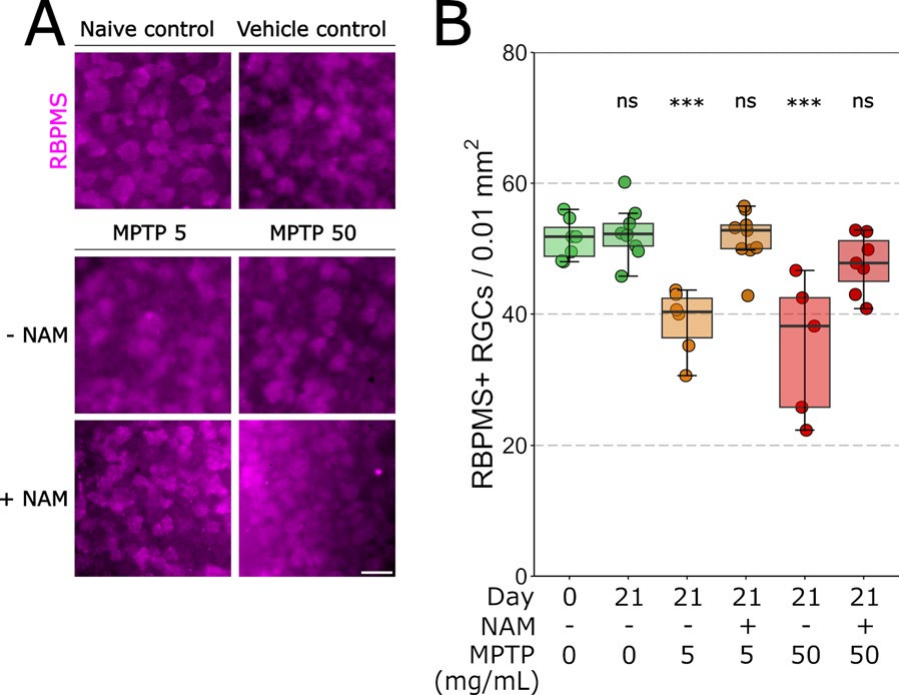
Nicotinamide provides a robust, long-term retinal ganglion cell neuroprotection following intravitreal MPTP administration. **A** Retinal ganglion cells were labelled in whole-mounted retina with anti-RBPMS (magenta) in Thy1-CFP mice. **B** There was no significant difference in RBPMS+ retinal ganglion cell density between the vehicle control group and the groups treated with NAM prior to injection of 5 mg/mL MPTP or 50 mg/mL MPTP, supporting the protection of retinal ganglion cells by NAM. Scale bar = 20 µm in A

NAM has previously been demonstrated to be robustly neuroprotective to retinal ganglion cells, in particular by protecting against mitochondrial dysfunction and injury [57, 68]. NAM has also been proposed as a treatment for Parkinson’s disease with numerous clinical trials ongoing [46]. We investigated the potential of NAM to provide neuroprotection against MPTP-induced retinal ganglion cell loss. Mice received oral NAM (500 mg/kg/days) from 7 days before MPTP injection till 21 days post-injection, then retinal ganglion cell survival was assessed (Fig. 5A). RBPMS+ retinal ganglion cell density was significantly lower in untreated retinas 21 days following MPTP injection than in naïve control retinas for 5 (Table 6,Fig. 5B) and 50 mg/mL (Table 6,Fig. 5B) of MPTP. In contrast, the retinas treated with NAM displayed no significant difference in RBPMS+ retinal ganglion cells compared to the naïve control treatment retinas (Table 6,Fig. 5B). These results demonstrate that NAM can protect against retinal ganglion cell neurodegeneration following intravitreal MPTP administration. CFP+ cells did not follow this result (Table S2; Figure S2A, B).

**Table 6:** *P* value and 95% CI per comparison of conditions in the nicotinamide treatment analysis

Nicotinamide treatment	*P* value	95% CI
RBMPS+ cell soma count		
Naïve control—vehicle control	0.995	[− 5.951, 7.934]
Naïve control—MPTP 5 mg/ml	< 0.001 ***	[− 20.232, − 4.903]
Naïve control—NAM+ MPTP 5 mg/ml	1.000	[− 6.719, 7.166]
Naïve control—MPTP 50 mg/ml	< 0.001 ***	[− 24.395, − 8.262]
Naïve control—NAM+ MPTP 50 mg/ml	0.546	[− 11.092, 3.635]

Significance: *P < 0.05, **P < 0.01, ***P < 0.001

## Discussion

There is a retinal phenotype in many Parkinson’s disease patients which is partially recapitulated in mouse models of the disease, including systemic MPTP administration. To assess this at the level of the retina without additional systemic complications or effects, mice were intravitreally injected with neurotoxin MPTP. Whilst there was no detectable loss of dopaminergic amacrine cell somas or dendritic integrity, there was a clear and reproducible loss of retinal ganglion cells in the inner retina.

We initially hypothesized that intravitreal injection of MPTP would drive dopaminergic amacrine cell dysfunction and possible degeneration. Dopaminergic neurons degenerate in the substantia nigra pars compacta and dopaminergic amacrine cells are lost in the retina of Parkinson's disease patients [24, 40]. In mouse models where MPTP is injected systemically, TH+ cell loss in the retina is variable and has been reported from 10 to 50 days post-injection [42, 54, 55]. However, these studies only report TH+ cell loss after repeated systemic MPTP injections, suggesting that multiple intravitreal injections may be necessary to cause TH+ cell loss in the retina. In goldfish eyes the cellular subtype composition and neurodegenerative/neuroprotective responses are markedly different to mammals. Here, TH+ cell and retinal content loss were reported as soon as 16 h up until 60 days after a single MPTP intravitreal injection of comparable MPTP dose (Villani 1988, Poli 1989, Villani 2000). To date, intravitreal MPTP administration in the mammalian eye has not been definitively tested. Whether this accumulated exposure is time-dependent or requires maintenance of high levels of MPTP in the vitreous to drive TH+ cell degeneration is unclear.

Unexpectedly, intravitreal MPTP administration did not result in the degeneration of TH+ retinal dopaminergic amacrine cells. When the amacrine cell integrity was analyzed, no significant differences could be identified following MPTP injection. While we cannot preclude changes to dopamine levels, the cell density of dopaminergic neurons in the mouse retina is very low (480 ± 40 dopaminergic neurons total per retina [58] compared to ~ 25.000 dopaminergic neurons in the rodent midbrain [14]), and as such any potential difference in retinal dopamine levels between the different groups would be difficult to detect. Our data identified the rapid neurodegeneration of retinal ganglion cells (~ 20% loss compared to controls). No significant loss of other retinal neurons or gross retinal thinning was observed, supporting retinal ganglion cell-specific neurodegeneration. MPTP is often compared to rotenone, another mitochondrial Complex I inhibitor routinely used for the modelling of Parkinson's disease. After the intravitreal injection of rotenone, a severe and acute reduction of retinal ganglion cells occurs (21% reduction 24 h post-injection of 1.2 mM of rotenone) [72]. The observed loss of retinal ganglion cells in this study is much less acute, supporting that MPTP can be used to induce a more chronic and less severe loss of retinal ganglion cells over time. Given the absence of systemic metabolism of MPTP and lack of gross neuroinflammation, we questioned the mechanism by which intravitreal MPTP might drive retinal ganglion cell loss. Analysis of gene expression in both mouse and human retina supports the potential of a mechanism where retinal ganglion cells metabolize MPTP to MPP+ internally via expression of *MAOB*. The identification of multiple different clusters of retinal ganglion cells in retina with differing *MAOB* expression suggests that there is no subtype which may be more susceptible to MPTP degeneration. However, this needs to be further investigated *e.g.* by use of mouse lines which express fluorescent fusion proteins marking the different retinal ganglion cell types. Given that in our model, MPTP is delivered to the vitreous, it seems most plausible that retinal ganglion cells are primarily affected as they occupy the most inner retinal layers (NFL/GCL) and could take up the lipophilic MPTP directly. MPTP may therefore not reach the inner retina in sufficient concentration to induce loss of TH+ amacrine cells which reside in the INL. While the inner limiting membrane and inner plexiform layer can represent significant diffusion barriers, this is in humans typically for compounds over 75 kDa and as such MPTP is unlikely to be restricted by these physical barriers [22]. In the mouse retina, expression of *Maob* is greater in vascular endothelium and fibroblasts than in retinal ganglion cells, with weaker expression in astrocytes and Müller glia [33]. When delivered systemically, MPTP would have access to all retinal layers via the 3 vascular plexuses and could be converted to MPP+ by fibroblasts, and Müller glia and retinal ganglion cells likely receive a lower concentration of MPTP than when delivered in the vitreous and cannot uptake MPP+ due to a lack of *Slc6a3* expression. However, when MPTP is injected in the vitreous we hypothesize that conversion in the toxic MPP+ happens in the retinal ganglion cells, due to *Maob* expression. Rat retinal ganglion cells have previously been found to express MAO-A [38], an enzyme which metabolizes a related toxin, 2'Me-MPTP, to MPP+ in mice [27]. People living with Parkinson’s disease have a reduction in the thickness of OCT layers that correspond to retinal ganglion cells including thinner retinal nerve fiber and ganglion cell inner plexiform layers [21, 62], a finding recapitulated in monkeys treated with systemic MPTP [50].

Neuroinflammation is likely to be an important component of Parkinson’s disease [28]. Systemic injection of MPTP has been reported to induce activation of microglia, astrocytes and Müller cells in the retina [6, 7, 41]. As MPTP is primarily metabolized to MPP+ by glial cells, we questioned whether intravitreal MPTP administration drives neuroinflammation that provides a pro-neurodegenerative environment and drives retinal ganglion cell neurodegeneration. There were no detectable changes to the retinal content of IBA1, GFAP, or GS supporting a lack of morphological change or gliosis of microglia, astrocytes and Müller cells in the retina. Future experiments such as quantification of cytokine expression would more definitively identify whether neuroinflammation occurs in this model. We only assessed inflammation 7 days following injection. Inflammation may be greater at later time points, and this remains to be determined. However, as retinal ganglion cell loss occurs early in this model, there is likely to be an uncoupling of any retinal ganglion cell degenerative event and a neuroinflammatory event. Our data do not preclude that inflammation is present at this 7 day timepoint, but the lack of a clear signature suggests at least that inflammation is not the driver of retinal ganglion cell loss at this time point.

Why are retinal ganglion cells vulnerable to intravitreal MPTP injection? MPP+ inhibits mitochondrial Complex I, III and IV resulting in mitochondrial and metabolic dysfunction [29, 35, 39]. Retinal ganglion cells are the susceptible neurons in many diseases characterized by mutations in mitochondrial genes or genes encoding mitochondrial or metabolic pathway proteins. Inherited optic neuropathies such as Leber hereditary optic neuropathy and autosomal dominant optic atrophy are driven by whole-body mutations to mitochondrial proteins, yet typically present with a retinal ganglion cell-only phenotype. In glaucoma, a neurodegenerative disease characterized by the death of retinal ganglion cells, mitochondrial and metabolic dysfunction are also key pathogenic mechanisms [56]. The high frequency of mitochondrial Complex I mutations [1], variation in mitochondrial transcription factor A [1] and thinning of the ganglion cell layers in Parkinson’s disease patients [8, 37, 21, 63] are suggestive of the potential for retinal ganglion cell loss in Parkinson’s disease.

As retinal ganglion cells are vulnerable to metabolic stress and treatments targeting these processes are neuroprotective [56], we next assessed whether administration of NAM (the amide of vitamin B_3_ and a precursor to the essential metabolite nicotinamide adenine dinucleotide (NAD), via drinking water accessible) could provide neuroprotection for dopaminergic amacrine cells in this intravitreal MPTP model. Metabolic dysfunction has gained increasing attention in the disease pathology of Parkinson’s disease and other common neurodegenerative diseases [45] with NAD deficiency frequently detected [64, 65, 71]. In our studies, administration of NAM resulted in robust protection of retinal ganglion cells for both the 5 and 50 mg/mL MPTP dose that lasted to the endpoint of the experiment at 21 days after injection. Elevating NAD through NAM, or increasing expression of NAD-generating enzymes, provides robust neuroprotection in experimentally induced (glaucomatous) retinal ganglion cell loss [57, 67, 68, 73]. NAM also provides robust neuroprotection against retinal ganglion cell loss from the potent Complex I inhibitor rotenone [57], which is also used to model Parkinson’s disease. In Parkinson's disease models restoring NAD pools has been demonstrated to ameliorate the disease phenotype. Pre-injection of NAD in the striatum of the 6-hydroxydopamine (a neurotoxin acting on mitochondrial Complex I) mouse model of Parkinson's disease ameliorated motor deficits and dopaminergic neuronal damage in the substantia nigra and striatum [51]. Additionally, in systemic MPTP models of Parkinson's disease, the administration of NAM resulted in the dose-dependent sparing of striatal dopamine levels and substantia nigra neurons [2], and also ameliorated Parkinsonian motor symptoms [48]. Parkinson’s disease clinical trials using NAM or other NAD-boosting agents are ongoing [46].

Our data demonstrate that a single intravitreal MPTP injection results in acute retinal ganglion cell death in the retina. The model is quick and retina-specific, and as such, does not induce a damaging systemic burden when only the retinal phenotype is of interest. Importantly, this acute degeneration is preventable allowing the model to be utilized for neuroprotection studies in the future. Despite this, the model lacks the recapitulation of the full spectrum of the retinal phenotype of Parkinson's disease. Electroretinograms could further elucidate the effect of MPTP on the function of retinal neurons and may reveal further deficits to the retina that are not identified by our structural measures. Future experiments could fully examine metabolic and neuroinflammatory phenotypes longitudinally, perhaps with repeated intravitreal injections to drive TH+ cell loss or a longer follow-up period. However, a higher or repeated administered dose of MPTP or a longer follow-up period could result in a pan-neuronal late-stage neurodegenerative phenotype, making it impossible to attribute TH+ cell death to MPP+ toxicity alone. Nonetheless, this model represents a useful expansion to the toolkit of scientists exploring neurodegeneration and metabolism.

## Conclusions

We have developed a model characterized by the specific loss of retinal ganglion cells with relevance to optic neuropathies and Parkinson’s disease. Importantly, NAM is neuroprotective in this model, supporting its use as a model for studying neuroprotection and the potential for NAM to be of use to a broad array of metabolic and neurodegenerative insults in the eye.

### Supplementary Information

**Additional file 1**. Supplementary Table 1, Supplementary Figure 1, Supplementary Figure 2. 

## Data Availability

All data generated or analyzed during this study are included in this published article.

## Abbreviation

MPTP: 1-Methyl-4-phenyl-1,2,3,6-tetrahydropyridine
MPP+: 1-Methyl-4-phenylpyridinium
DAPI: 4′,6-Diamidino-2-phenylindole
ATP: Adenosine triphosphate
BSA: Bovine serum albumin
CI: Confidence interval
CFP: Cyan fluorescent protein
GCL: Ganglion cell layer
GFAP: Glial fibrillary acidic protein
GS: Glutamine synthetase
GFP: Green fluorescent protein
HBSS: Hank’s balanced salt solution
IPL: Inner plexiform layer
IBA1: Ionized calcium-binding adaptor molecule 1
MAO-B: Monoamine oxidase B
NFL: Nerve fiber layer
NAM: Nicotinamide
NAD: Nicotinamide adenine dinucleotide
ONL: Outer nuclear layer
OPL: Outer plexiform layer
PFA: Paraformaldehyde
PBS: Phosphate-buffered saline
Prox1: Prospero-related homeobox 1
RBPMS: RNA-binding protein with multiple splicing
