# Supplementary material for: Intravitreal MPTP drives retinal ganglion cell loss with oral nicotinamide treatment providing robust neuroprotection
Source: Acta Neuropathol Commun. 2024 May 21;12:79. doi: 10.1186/s40478-024-01782-3 (PMC11107037; doi:10.1186/s40478-024-01782-3)
Supplement: Supplementary file 1 — Additional file 1. Supplementary Table 1, Supplementary Figure 1, Supplementary Figure 2. [file 40478_2024_1782_MOESM1_ESM.doc]

**Supplementary material**

**Results**

*Intravitreal MPTP administration does not result in TH+ cell loss*

| CFP+ cell soma count |  |  |
| --- | --- | --- |
| Naïve control – vehicle control | 0.151 | - |
| Naïve control – MPTP 5 mg/ml 7 days after injection | < 0.001 *** | [-15.882, -3.839] |
| Naïve control – MPTP 5 mg/ml 14 days after injection | 0.004 ** | [-15.997, -2.702] |
| Naïve control – MPTP 5 mg/ml 21 days after injection | < 0.001 *** | [-17.869, -4.574] |
| Naïve control – MPTP 50 mg/ml 7 days after injection | 0.010 ** | - |
| Naïve control – MPTP 50 mg/ml 14 days after injection | 0.010 ** | - |
| Naïve control – MPTP 50 mg/ml 21 days after injection | 0.010 ** | - |

**Table S1. P-value and 95% CI per comparison of conditions in the retinal ganglion cell analysis.** Significance: * = P < 0.05, ** = P < 0.01, *** = P < 0.001.

**
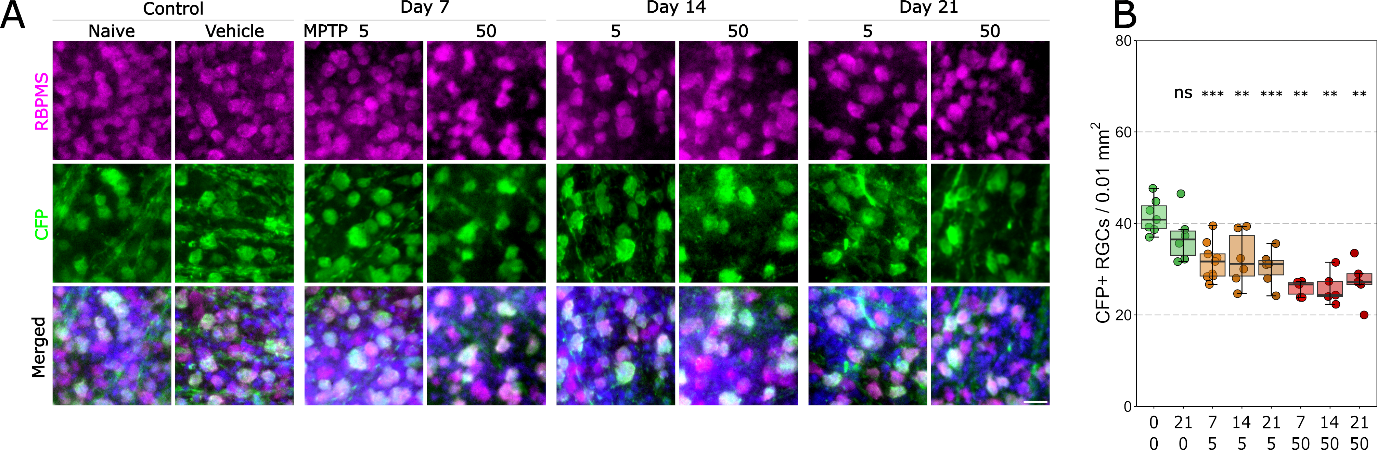
**

**Figure S1. Intravitreal MPTP administration drives retinal ganglion cell loss.** (**A**) Retinal ganglion cells were fluorescently labelled in whole-mounted retina from Thy1-CFP mice with anti-RBPMS (magenta) and anti-GFP (green). (**B**) There were no significant differences between the naïve and vehicle control (green) groups. CFP+ retinal ganglion cell density was not significantly different to vehicle control following 5 mg/mL MPTP (orange) at 7, 14 and 21 days post-injection but was significantly decreased at these time points with 50 mg/mL of MPTP.

*Nicotinamide provides robust, long-term retinal ganglion cell neuroprotection following intravitreal MPTP administration*

| CFP+ cell soma count |  |  |
| --- | --- | --- |
| Naïve control – vehicle control | 0.281 | [-11.339, 2.196] |
| Naïve control – MPTP 5 mg/ml | < 0.001 *** | [-17.989, -4.454] |
| Naïve control – NAM + MPTP 5 mg/ml | < 0.001 *** | [-17.652, -4.648] |
| Naïve control – MPTP 50 mg/ml | < 0.001 *** | [-21.427, -7.182] |
| Naïve control – NAM + MPTP 50 mg/ml | 0.020 * | [-13.930, -0.931] |

**Table S2. P-value and 95% CI per comparison of conditions in the retinal ganglion cell analysis.** Significance: * = P < 0.05, ** = P < 0.01, *** = P < 0.001.


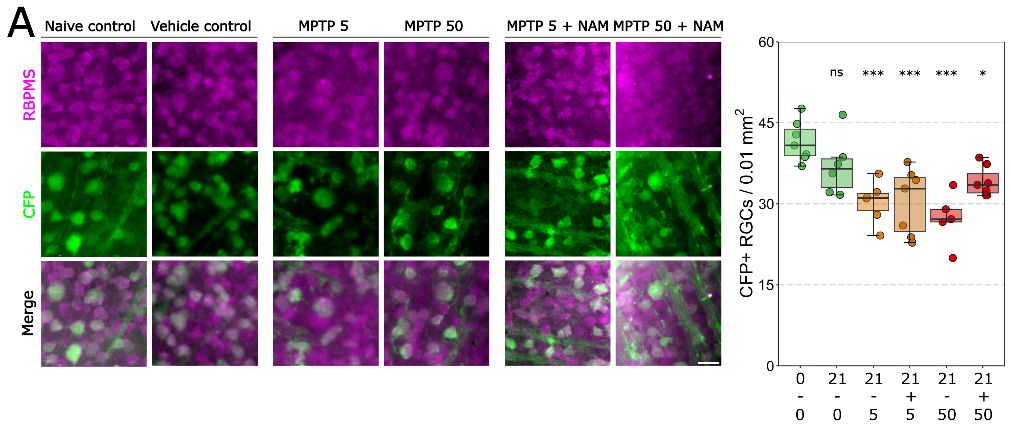


**Figure S2 Nicotinamide provides a robust, long-term retinal ganglion cell neuroprotection following intravitreal MPTP administration.** (**A**) Retinal ganglion cells were labelled in whole-mounted retina with anti-RBPMS (magenta) and anti-GFP (green) in Thy1-CFP mice. (**B**) There was no significant difference in CFP+ retinal ganglion cell density between the vehicle control group and the groups treated with NAM prior to injection of 5 mg/mL MPTP or 50 mg/mL MPTP, supporting the protection of retinal ganglion cells by NAM. Scale bar = 20 µm in A.
